# Cryopreserved cGMP-compliant human pluripotent stem cell-derived hepatic progenitors rescue mice from acute liver failure through rapid paracrine effects on liver cells

**DOI:** 10.1186/s13287-024-03673-9

**Published:** 2024-03-12

**Authors:** Malika Gantier, Raphaël Rispal, Angélique Fourrier, Séverine Ménoret, Frédéric Delbos, Ignacio Anegon, Tuan Huy Nguyen

**Affiliations:** 1 GoLiver Therapeutics, 44007 Nantes, France; 2 Nantes Université, Inserm, Center for Research in Transplantation and Translational Immunology, UMR 1064, 44000 Nantes, France; 3 Nantes Université, CHU Nantes, Inserm, CNRS, SFR Santé, Inserm UMS 016 CNRS UMS 3556, 44000 Nantes, France

**Keywords:** Regenerative medicine, Pluripotent stem cells, Hepatic progenitor cells, Acute liver failure, Acetaminophen, Thioacetamide, cGMP, Cryopreservation

## Abstract

**Background:**

Liver transplantation remains the only curative treatment for end-stage liver diseases. Unfortunately, there is a drastic organ donor shortage. Hepatocyte transplantation emerged as a viable alternative to liver transplantation. Considering their unique expansion capabilities and their potency to be driven toward a chosen cell fate, pluripotent stem cells are extensively studied as an unlimited cell source of hepatocytes for cell therapy. It has been previously shown that freshly prepared hepatocyte-like cells can cure mice from acute and chronic liver failure and restore liver function.

**Methods:**

Human PSC-derived immature hepatic progenitors (GStemHep) were generated using a new protocol with current good manufacturing practice compliant conditions from PSC amplification and hepatic differentiation to cell cryopreservation. The therapeutic potential of these cryopreserved cells was assessed in two clinically relevant models of acute liver failure, and the mode of action was studied by several analytical methods, including unbiased proteomic analyses.

**Results:**

GStemHep cells present an immature hepatic phenotype (alpha-fetoprotein positive, albumin negative), secrete hepatocyte growth factor and do not express major histocompatibility complex. A single dose of thawed GStemHep rescue mice from sudden death caused by acetaminophen and thioacetamide-induced acute liver failure, both in immunodeficient and immunocompetent animals in the absence of immunosuppression. Therapeutic biological effects were observed as soon as 3 h post-cell transplantation with a reduction in serum transaminases and in liver necrosis. The swiftness of the therapeutic effect suggests a paracrine mechanism of action of GStemHep leading to a rapid reduction of inflammation as well as a rapid cytoprotective effect with as a result a proteome reprograming of the host hepatocytes. The mode of action of GStemHep relie on the alleviation of inhibitory factors of liver regeneration, an increase in proliferation-promoting factors and a decrease in liver inflammation.

**Conclusions:**

We generated cryopreserved and current good manufacturing practice-compliant human pluripotent stem cell-derived immature hepatic progenitors that were highly effective in treating acute liver failure through rapid paracrine effects reprogramming endogenous hepatocytes. This is also the first report highlighting that human allogeneic cells could be used as cryopreserved cells and in the absence of immunosuppression for human PSC-based regenerative medicine for acute liver failure.

**Supplementary Information:**

The online version contains supplementary material available at 10.1186/s13287-024-03673-9.

## Background

Acute liver failure (ALF) is a rare and severe liver disease that has a high mortality and affects many organ systems. It is characterized by a rapid and massive deterioration of liver functions, which results in hepatic encephalopathy and coagulopathy within 8 weeks of the first symptoms in individuals with no preexisting liver disease [1, 2]. The main causes of this critical pathology are drug-induced liver injury, intoxication, viral hepatitis, autoimmune liver disease and shock or hypoperfusion [1, 3].

So far, orthotopic liver transplantation (OLT) is the only curative treatment for severe forms of ALF at the end stage. Unfortunately, because of a drastic shortage of organ donors, many patients are excluded from the liver waiting list with exclusion criteria such as cancer outside the liver, substance abuse, disabling psychiatric conditions or lack of inadequate insurance. In addition, this treatment requires a heavy surgery with higher mortality risks for elderly patients and/or patients with preexisting cardiac illness and a lifelong immunosuppressive treatment with known side effects such as infections, renal toxicity and a higher risk of cancer [4].

Hepatocyte transplantation (HT) isolated from discarded livers for transplantation due to quality criteria has become an attractive alternative or a bridge to OLT [5–7]. This alternative has been tested in patients with ALF or inherited metabolic diseases such as Crigler-Najjar syndrome type 1, urea cycle defects or factor VII deficiency [8]. HT has demonstrated positive results by improving patients' liver functions with decreased blood bilirubin levels, spontaneous healing, or successful transition to liver transplantation. However, the results obtained from these studies could not define the exact conditions and population of patients for which this therapy would be beneficial [8, 9]. This can be explained by the limited number of patients, the variable quality of the isolated hepatocytes, and the different protocols used between care centers. Moreover, current culture conditions fail to efficiently expand primary hepatocytes in vitro to a sufficient scale for the treatment of many patients, resulting in a continual need for multiple liver donors [10–12]. HT has paved the way to cell therapy as an alternative to OLT, but a new source of cells, available of constant quality at any time and on a large scale, is needed.

Pluripotent stem cells (PSC) have emerged as the ideal cell source. Indeed, they have the capacity for unlimited self-renewal and to differentiate into any cell type of the body, including hepatocytes. Many teams have already developed protocols for the differentiation of PSCs into hepatocytes, so-called hepatocyte-like cells (HLC), which do not have a fully mature hepatic phenotype, as they still express alpha-fetoprotein (AFP), a marker of fetal hepatocytes [13–17]. The therapeutic potential of HLCs has been demonstrated in ALF animal models using freshly prepared cells and non-compliant current good manufacturing practice (cGMP) conditions, e.g., due to the use of Matrigel as an extracellular culture matrix [18–22]. While these results are encouraging, HLC transplantation in humans presents serious challenges. Indeed, similar to primary hepatocytes, these cells might be highly sensitive to cryopreservation and lose most of their therapeutic potential upon cell thawing [23, 24]. However, it is very important to cryopreserve the cells to always guarantee the availability of treatment at any time in the care centers. While cell cryopreservation is a key point for the emergency treatment of patients with end-stage liver diseases, only freshly prepared HLCs were used for treating ALF animal models. We have previously shown that cryopreserved PSC-derived hepatic cells can treat an inherited metabolic liver disease, but this is a pathological context where hepatic cells have time to engraft and to mature into fully differentiated hepatocytes [25]. Moreover, the current production protocols must comply with cGMP to be used as medicine in the clinic. Few studies have presented cGMP-compliant protocols, and none have evaluated them for the treatment of animals modeling acute or chronic liver diseases using cGMP-compliant HLCs [26]. In addition, as previously shown in humans, there is poor integration of transplanted cells in the host liver despite immunosuppression, suggesting a poor understanding of the mechanisms leading to failure of cell implantation and/or cell rejection as well as a lack of efficient ways to prevent it [27–29].

Here, we describe the production, cryopreservation and characterization of human PSC-derived hepatic progenitors called GStemHep generated using cGMP-compliant protocols. These cells present an immature hepatic phenotype (positive for AFP, negative for albumin/ALB), secrete hepatocyte growth factor (HGF) and have a low immunogenic profile (no expression of major histocompatibility complex (MHC) class I or II). We show the therapeutic effect of cryopreserved GStemHep in vivo in two different models of ALF, i.e., acetaminophen (APAP) overdose in immunodeficient mice (NOD/SCID) and thioacetamide (TAA) overdose in immunocompetent mice (C57Bl/6) in the absence of immunosuppression. We show that cryopreserved cGMP-compliant GStemHep rescue mice from ALF with a better understanding of the mechanism of action and its kinetics following cell transplantation. Indeed, GStemHep lead to a decrease in APAP-induced inflammation and the restoration of liver functions. We have therefore overcome the current challenges in liver cell therapy with unlimited cell production from the same starting cell raw material (human PSC) to avoid variable quality of cell therapy products due to multiple donor sourcing (inherent inter-individual variability), cGMP-compliant therapeutic cells (PSC amplification and hepatic differentiation) and cryopreserved cells for immediate clinical availability.

## Methods

### Cell culture

The human PSC cell line was provided by ESI BIO (Alameda, USA) and derived under cGMP conditions on human fibroblast feeder layers [30]. They were adapted and then cultured in feeder-free conditions on culture dishes coated with the vitronectin recombinant human protein (Thermo Fisher) in mTeSR1™ medium (StemCell Technologies) at 37 °C in a 5% CO_2_ incubator with daily media changes and were passaged every 4–5 days using TrypLE™ (Thermo Fisher), following the manufacturer’s recommendations. After each passage, they were cultured for 24 h in the presence of 10 μM of the Rock inhibitor Y-27632 (StemCell Technologies).

### In vitro new hepatic differentiation protocol and GStemHep culture

**Fig. 1:**
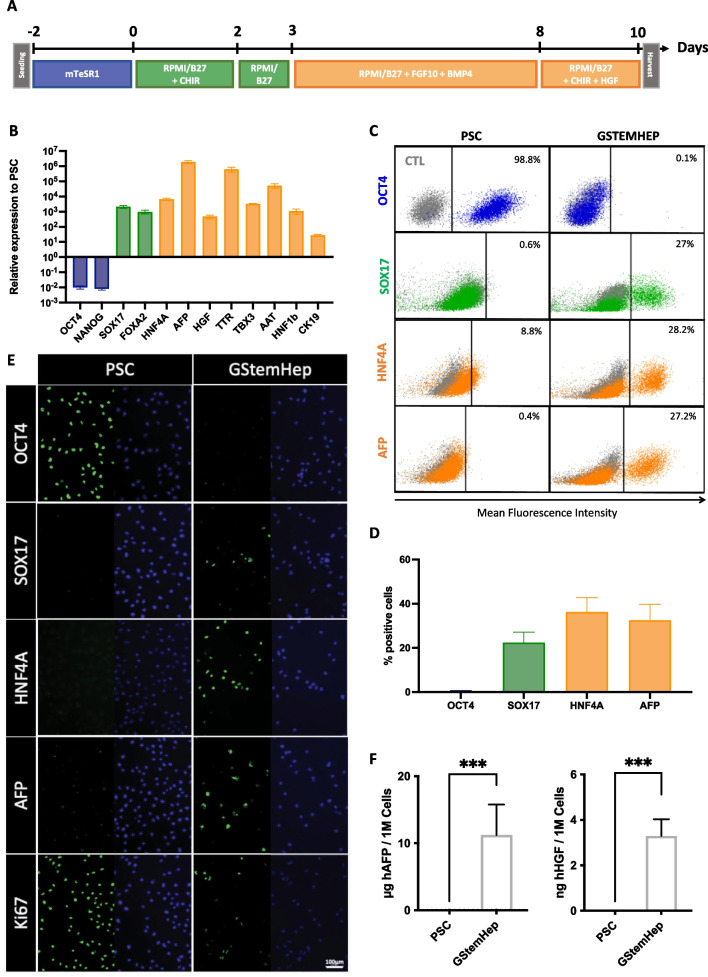
Expression profile of differentiation markers in GStemHep cells. **A** Schematic diagram of PSC-derived GStemHep cells. Human PSC were differentiated into GStemHep cells with a 10-day protocol. The expression profile of the indicated markers was analyzed in cells and culture supernatants. Pluripotency, endoderm and hepatic markers are shown in blue, green and orange, respectively (except for immunofluorescence staining). **B** RT‒qPCR analysis of the expression of key markers for differentiation stages and commitment to liver fate in GStemHep (n = 10). The results were normalized to the GAPDH housekeeping gene and expressed as the fold change relative to PSC. **C** Representative FACS dot plot of a PSC and a GStemHep production batch (detection threshold of OCT4 flow cytometry is 0.2%). Cells incubated with isotype control are in gray. **D** Average expression of pluripotency factor (OCT4) and hepatoblast markers (SOX17/HNF4A/AFP) by GStemHep (n = 9). **E** Immunofluorescence staining of GStemHep and human PSC for pluripotency (OCT4), proliferation (Ki67), endoderm (SOX17) and hepatic markers (HNF4A et AFP). The marker proteins and DAPI are shown in green and blue, respectively (magnification 20x). **F** Detection of secreted human AFP and human HGF in culture supernatant by ELISA (n = 10) (***p = 0.0007 Mann‒Whitney test)

All reagents were GMP compliant. PSCs were plated onto iMatrix-511 (Amsbio) in mTeSR1™ with 10 μM of Rock inhibitor Y-27632, following the manufacturer’s recommendations, in two-dimensional culture systems. After 48 h, PSC maintenance medium was replaced with RPMI-1640 supplemented with B27 serum-free supplement (Life Technologies) to start differentiation (Day 0). During the first two days of definitive endoderm induction, cells were cultured in the presence of 3 μM CHIR-99021 (Stem Cell Technologies). Then, the cells were cultured for 1 day without cytokines. To induce hepatic cell specification, medium was supplemented with 10 ng/mL fibroblast growth factor 10 (FGF-10) (Miltenyi Biotec) and 10 ng/mL bone morphogenetic protein 4 (BMP-4) (R&D System) for five days. Finally, the cells were cultured in the presence of 3 μM CHIR-99021 and 20 ng/mL HGF (Miltenyi Biotec) for two days to obtain GStemHep (Fig. 1A). After differentiation, cells were harvested and frozen at − 150 °C in CryoStor® CS10 cell freezing medium (StemCell Technologies). Cell count and viability were evaluated using NC-200 NucleoCounter (Chemometec). For the plating experiments, GStemHep cells were thawed and seeded onto iMatrix-511-coated culture dishes in RPMI-1640 supplemented with B27, 10% FBS, 10 μM Y-27632, 3 μM CHIR-99021 and 20 ng/mL HGF.

### Animals

All animal care and procedures performed in this study were approved by the Animal Experimentation Ethics Committee of Pays de la Loire region, France. Male NOD/SCID mice and C57Bl/6 mice were obtained from Janvier Labs (Saint-Berthevin, France) and maintained in the animal facility on 12 h light/dark cycles with food and water ad libitum with sizzle paper enrichment. After receipt, mice were randomized for treatment and control groups and acclimatized for 10 days before experiment. Confounders were not controlled and experiments were performed as a non-blinded approach. Sample size was estimated based on our experience [25]. For all surgical procedures, mice were placed in an induction box until sufficiently anaesthetized with isoflurane (2–3% inhalation with oxygen). Once the desired depth was reached, mice were carefully placed on a heating pad and a respirator during surgery to maintain body temperature at 37 °C and anesthesia, respectively. Sterile lubrication gel was applied into the mice’s eyes to prevent corneal burns from the isoflurane. Mice that died during surgery (approximatively 5%) were excluded from the study. Weight loss and clinical signs of pain were set as the end points before the planned sacrifice.

### APAP-induced ALF model

Male NOD/SCID mice (6 weeks old) were treated with an intraperitoneal (ip) injection of 650 mg APAP/kg (Sigma‒Aldrich) to induce ALF. Three hours later, animals received an intrasplenic injection of 1 × 10^6^ cryopreserved GStemHep cells, which were thawed, pelleted by low-speed centrifugation and resuspended in RPMI/B27 medium. The control mice received APAP but no cell treatment. In one experiment, we used 1 × 10^6^ cryopreserved primary human hepatocytes (Biopredic International) as comparative therapeutic cells. Mice survival was followed over 10 days, or mice were euthanized by cervical dislocation at 6 h, 9 h and 24 h post-APAP.

### TAA-induced ALF model

Male C57Bl/6 mice (6 weeks old) were treated with an ip injection of 1500 mg TAA/kg (Sigma‒Aldrich) to induce ALF. Twenty-four hours later, animals received an intrasplenic injection of 1 × 10^6^ cryopreserved GStemHep, which were thawed and directly used for cell injection. The control mice received TAA but no cell treatment. Mice survival was followed over 7 days, or the mice were euthanized by cervical dislocation at 24 h.

### RNA extraction and real-time quantitative polymerase chain reaction (RT‒qPCR)

Total mRNA was extracted from cell culture using the RNeasy Mini kit (Qiagen) following the manufacturer’s recommendations. Total RNA from frozen liver was extracted using TRIzol (Life Technologies) and chloroform (VWR) after mechanical disruption of the tissue. Then, the RNeasy Mini kit was used for cell culture mRNA. RT‒qPCR was performed with 5 ng of mRNA, with a one-step RT‒PCR kit using Taqman® technology (AgPath-IDTM One-Step RT‒PCR, Life Technologies) and using the Applied Biosystems ViiA 7 Real-Time PCR System and the appropriate primers for Taqman assays (Life Technologies; Additional file 1: Table S1). The relative gene expression was calculated using the 2^−ΔΔCt^ quantification method after normalization to GAPDH values and expressed as fold of levels found in undifferentiated hPSCs cells for cultured cell or in wild type (WT) mouse group for liver analysis.

### Flow cytometry

Cryopreserved GStemHep cells were thawed, centrifuged and incubated on ice with fixable viability dye eFluor™ 450 (eBioscience) for 20 min. Intracellular staining was carried out according to the manufacturer’s instructions using a fixation/permeabilization kit (eBioscience) in the presence or absence of antibodies against OCT3/4 (OCT4) (AlexaFluor 647 mouse anti-OCT3/4, BD Biosciences), SOX17 (APC goat anti-SOX17, R&D system), AFP (AlexaFluor 647 mouse anti-AFP, R&D system) and HNF4A (Alexa Fluor 488 mouse anti-HNF4A, Santa Cruz). For membrane staining, antibodies against HLA-ABC (FITC mouse anti-HLA-ABC, BD Biosciences) and HLA-DR (APC mouse anti-HLA-DR, BD Biosciences) were used. Flow cytometry (FACS) analysis was performed with a FACSCanto II (BD Biosciences) and Flow Jo software (Tree star, Ashland, OR, USA).

### Enzyme-linked immunosorbent assay (ELISA) analysis

The quantity of human AFP and HGF secreted into the cell culture medium was determined using the AFP human ELISA Kit (Abnova) and the HGF human ELISA Kit (Invitrogen) following the manufacturer’s instructions. Human AFP secreted into the sera of animals was determined by the human AFP Elisa Quantification Kit (Invitrogen, EHAFP) following the manufacturer’s instructions.

### Immunofluorescence assay

Cultured cells were fixed with 4% paraformaldehyde for 15 min at room temperature, permeabilized with 0.5% Triton X-100 in PBS for 15 min and blocked with 1% BSA-0.1% Triton in PBS for 30 min. Primary antibodies were diluted in blocking solution and incubated for 1 h at room temperature. Secondary antibodies were diluted in blocking solution and incubated for 1 h at room temperature (primary and secondary antibody information in Additional file 2: Table S2). Cells were mounted using coverslips and ProLong Gold Antifade Mountant (Life Technologies). All pictures were observed under a Zeiss fluorescence microscope.

### Biochemical analysis

Liver injury was estimated by the increase in the serum activities of alanine-amino-transferase (ALAT) and aspartate-amino-transferase (ASAT) measured by the biochemicals laboratory of University Hospital Center (Nantes, France).

### Detection of the human Alu DNA sequence by PCR

Genomic DNA was extracted from the liver, spleen and lungs using Genomic DNA from Organs and Cells Kit (Macherey–Nagel) following the manufacturer’s recommendations. Alu PCR was conducted using two primers: hAluR: 5'-TTT TTT GAG ACG GAG TCT CGC TC-3' (SEQ ID NO: 1) and hAluF: 5'-GGC GCG GTG GCT CAC G-3' (SEQ ID NO: 2). PCR was carried out with a Herculase Kit (Agilent) in a total volume of 25 μL with 10 ng of genomic DNA. PCR cycling parameters were taken from the manufacturer's instructions. PCR products were run on a microfluidic 5 K chip in a capillary electrophoresis device (Caliper Labchip-GX, LifeScience).

### Histological analysis

Liver tissues were fixed in 4% paraformaldehyde and embedded in paraffin. Sections of 4 μm were used for hematoxylin-eosin-safran (HES) and immunohistochemistry (IHC) staining. After dewaxing, the paraffin slices were incubated for 20 min with EDTA buffer (10 mM; pH 9.0) or citrate buffer (10 mM; pH 6.0) at 98 °C, and endogenous peroxidases were blocked for 10 min using 3% H2O2. The presence of human cells was analyzed by specific detection of the human Ku80 protein in mouse livers. For this, slices were incubated for 45 min in Blocking Solution (Vector Laboratories), then with primary rabbit monoclonal anti-human Ku80 antibody (Abcam) for 1 h and finally with EnVision + Dual Link system-HRP (DAB +) (Agilent Dako) for 30 min at room temperature. For Ki67 staining, slices were sequentially incubated in avidin–biotin blocking solution (Invitrogen) for 10 min, in Animal-Free Blocker (Vector Laboratories) for 45 min and with primary rabbit polyclonal anti-mouse/human Ki67 antibody (Abcam) overnight at 4 °C. Afterwards, a rabbit-specific HRP/DAB (ABC) Detection IHC Kit (Abcam) was used according to the manufacturer’s manual. All IHC staining was counterstained with Mayer’s hematoxylin (Sigma‒Aldrich). After dehydration, slices were embedded in NeoMount medium (Merck Millipore) and scanned with a digital slide scanner (Nanozoomer, Hamamatsu Photonics). Files were analyzed with the NDP viewer 2.5 software (Hamamatsu).

### Proteomics

An unbiased quantification of all detectable peptides and proteins was performed in GStemHep supernatant and also in liver tissue of no APAP, APAP only and APAP with treatment mice by HRM™ mass spectrometry (Biognosys). For liver tissue analyses, differentially regulated proteins in each cluster/group were identified using the following criteria: q-value < 0.025 and average fold change > 1.5. Principal component analysis was conducted in R using *prcomp* and a modified *ggbiplo*t function for plotting, and partial least squares discriminant analysis was performed using *mixOMICS* package. Kyoto Encyclopedia of Genes and Genomes (KEGG) [31] and Gene Ontology (GO) [32] pathway annotations were performed using g:Profiler and String-db (string-db.org) [33].

### Statistical analysis

Statistical analysis was achieved with GraphPad Prism 5 software. All results are expressed as the mean ± SEM. Mann‒Whitney, Kruskal‒Wallis, and one- or two-way ANOVAs were used when appropriate. Animal survival was analyzed by means of the log rank (Mantel‒Cox) test.

## Results

### Differentiation of GStemHep using a cGMP-compliant protocol

We modified our previous protocol [25] to develop fully cGMP‐compliant production procedures for both human PSC maintenance/amplification and hepatic differentiation under feeder-free conditions using only small molecules, recombinant factors and GMP-available reagents and cell culture media. At the end of the hepatic differentiation process, cell supernatants were kept for the measurement of secreted molecules, and GStemHep cells were frozen for various RNA and protein analyses. The cell identity and gene expression profile were first analyzed by RT‒qPCR (Fig. 1B). The pluripotency-associated markers OCT4 and NANOG were lost in GStemHep, while the endoderm-associated genes FOXA2 and SOX17 and the fetal hepatocyte or hepatic progenitor-associated genes HNF4A, AFP, TBX3, TTR, AAT, HNF1b and CK19 were detected, as previously described by others [15]. We also detected the expression of HGF in GStemHep cells. The detected gene expression profile was validated at the protein level by flow cytometry analyses of harvested and frozen cells (Fig. 1C). On day 0 of hepatic differentiation, OCT4 is highly expressed in PSC and is lost after differentiation into GStemHep cells. The average expression of SOX17, HNF4A and AFP markers in GStemHep cells was 22.3% (± 4.8), 36.2% (± 6.5) and 32.5% (± 7.2), respectively (Fig. 1D). All these results were corroborated by an immunofluorescence assay with a complete loss of expression of the pluripotency OCT4 and gain of hepatic-specific gene markers SOX17, HNF4A and AFP in GStemHep while PSC were highly positive for OCT4 and negative for AFP and HNF4A (Fig. 1E). Interestingly, GStemHep maintained some proliferative activity, as assayed by immunostaining of the cell cycle-associated marker Ki67, while as expected, PSC were highly proliferative (Fig. 1E). GStemHep secreted AFP and HGF in the cell culture supernatants at the level of 11.2 µg/10^6^ cells and 3.3 ng/10^6^ cells during the last 24 h of differentiation, as detected by ELISA, respectively (Fig. 1F). The secretion of these two molecules by GStemHep cells was confirmed by proteomic mass spectrometry analysis of the cell supernatant (not shown). As expected, no AFP or HGF was detected in the undifferentiated PSC cell supernatant. GStemHep do not express mature hepatocyte-specific markers, such as albumin and CYP3A4 cytochrome P450 (not shown). In addition, GStemHep did not show expression of MHC class II (HLA-DR molecule) and lost the expression of MHC class I (HLA-ABC molecule, 2.3% ± 0.6), which was initially detected in PSC (92.4%), as assessed by flow cytometry (Additional file 3: Fig. S1).

In conclusion, we developed a fully compatible process to produce GStemHep cells that have the phenotype of immature hepatic progenitor cells (AFP + /ALB−) secreting human AFP and HGF with some proliferative activity.

### Cryopreservation of GStemHep

**Fig. 2:**
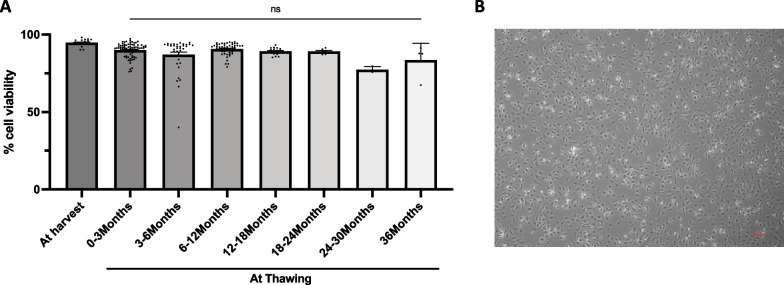
Cryopreservation of GStemHep. After 10 days of differentiation, GStemHep cells were harvested and frozen in a cryopreservation solution. Cell viability and adhesion capacity were analyzed after thawing. **A** Viability of freshly produced GStemHep at harvest and thawing after different months of freezing at − 150 °C; each dot represents a different batch or thawing (n = 202) (Kruskal‒Wallis test, ns: not significant). **B** Morphology of GStemHep (1 year frozen) 24 h after thawing and seeding onto a culture plate (Magnification 5x)

To use GStemHep for cell therapy as frozen cells, we evaluated several cGMP-compliant cryopreservation solutions. Here, GStemHep cells were cryopreserved in CryoStor® CS10 cell freezing medium and stored at − 150 °C. The cells were then thawed to analyze the impact of freezing on their viability and their replating ability after different time periods of cryopreservation. The viability of harvested and freshly produced GStemHep was 94.7% ± 0.5 (average of 18 production batches) (Fig. 2A). The cell viability was not changed in our cryopreservation and storage conditions over time for up to 36 months, as shown in Fig. 2A. The viability of frozen cells was 83.5% (± 5.4) after 36 months of storage, which was not significantly changed compared to 89.9% (± 0.5) at 0–3 months of storage. We showed that cryopreserved GStemHep were able to attach onto culture dishes after thawing, showing that they retained their functional adhesive properties to the recombinant laminin-extracellular matrix for up to 12 months (Fig. 2B). Maintenance of the immature hepatocyte phenotype of cryopreserved GStemHep was also evaluated by FACS and RT‒qPCR analysis on one production. In both analyses, no difference was observed between fresh and thawed cells in the expression of pluripotency (loss), endoderm or hepatic markers (gain) (Additional file 4: Fig. S2).

To conclude, the overall freezing conditions of GStemHep were optimized for a high viability after thawing with the maintenance of the cell phenotype and the adhesion capacities, equivalent to that of the freshly produced cells for a long period storage.

### Frozen GStemHep rescue APAP-induced acute liver failure in T-cell immunodeficient mice

**Fig. 3:**
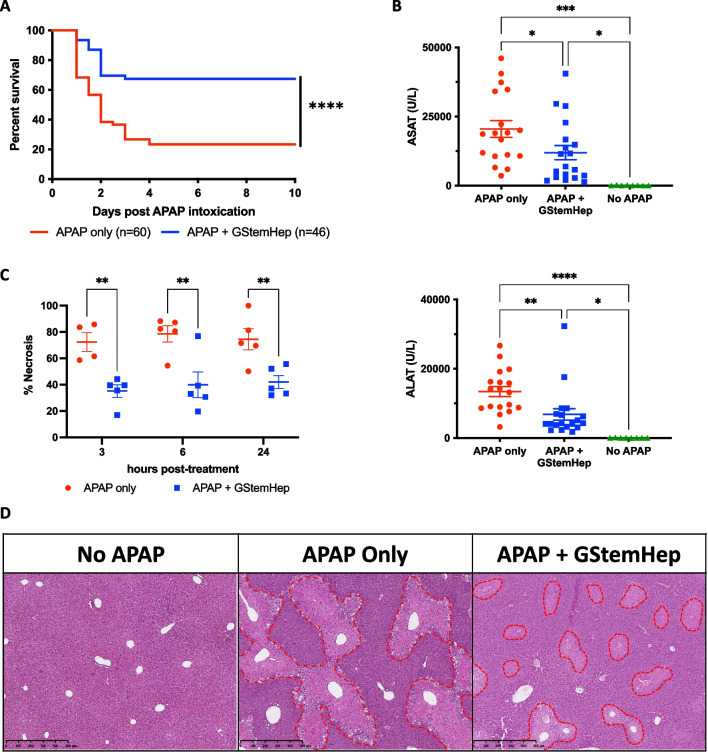
Therapeutic effect of GStemHep in NOD/SCID mice with APAP-induced acute liver failure. After APAP intoxication, mice were treated (APAP + GStemHep) or not (APAP only) with intrasplenic injection of 1 × 10^6^ thawed GStemHep. Mouse survival was followed over 10 days, or mice were euthanized at 3 h, 6 h and 24 h post treatment for analysis of liver damage markers. **A** Survival curve of APAP-induced ALF mice treated with GStemHep injection (n = 46) compared with non-treated group (n = 60) (****p < 0.0001 log-rank (Mantel‒Cox) test). **B** Biochemical analysis of liver damage markers: ASAT (up) and ALAT (down) in the serum of each group (n = 18 for APAP only, n = 19 for APAP + GStemHep and n = 8 for no APAP) 24 h after APAP intoxication (*p < 0.05; **p < 0.005; ***p < 0.0005; ****p < 0.0001 one-way ANOVA test). **C** Necrosis quantification on HES-stained liver sections in APAP only (n = 5) and APAP + GStemHep groups (n = 5) at 3 h, 6 h and 24 h after GStemHep transplantation (**p < 0.01 two-way ANOVA test). **D** Representative HES-stained liver sections from each group (n = 5 per group) 24 h after GStemHep transplantation (magnification 5x). Areas of necrosis are delimited by the red dotted lines

To investigate the therapeutic potential of frozen GStemHep for acute liver failure, we used a model of ALF in NOD/SCID-T-cell-deficient mice lethally intoxicated with APAP, as previously described [20]. After APAP overdose, 80% of animals died within 4 days, with > 50% lethality occurring within 2 days (Fig. 3A). In contrast, after a unique dose of frozen GStemHep (1 × 10^6^ cells) that was thawed and injected into the liver via the spleen, most ALF mice survived, which was significantly different from untreated control mice (*p* < 0.05) (Fig. 3A). Notably, this therapeutic benefit was obtained with different cell production batches (n = 5) cryopreserved for up to 5 months and in different animal studies (n = 8, total of 46 treated mice). We have also shown in a comparative study that the therapeutic effect of GStemHep was greater than that of primary hepatocytes (100 vs. 60% of survival), although the difference was not statistically significant because of the animal group size was too small (Additional file 5: Fig. S3).

The increase in survival was accompanied by a significant decrease in the markers of liver damage ASAT and ALAT in the serum of the treated animals at 24 h post-injection (Fig. 3B). Noteworthy, this decrease was also observed at 3 h and 6 h after GStemHep injection (Additional file 6: Fig. S4A). The cytoprotective effect of GStemHep was also demonstrated by histopathological comparison of liver tissue. Massive necrosis (> 70%) is present in the liver as early as 3 h after intoxication, i.e., at the time of GStemHep injection (Additional file 6: Fig. S4B). At 3 h post-cell injection, there was a significant decrease in liver necrosis areas in the APAP-control group versus the GStemHep-treated group (72.5% ± 7.1 vs. 35.3% ± 4.8, *p* < 0.05, respectively) (Fig. 3C). Three hours later, i.e., 6 h post-cell injection, this reduction was maintained with a necrosis of 39.9% ± 9.8 in GStemHep-treated mice against 78.7% ± 6.2 (*p* < 0.01) in the APAP control group. Twenty-four hours after intoxication, massive liver necrosis was still histologically observed in the livers of untreated control ALF animals (Fig. 3D, middle photo) in contrast to GStemHep-treated ALF animals (Fig. 3D, right photo). As expected, no necrosis was observed in the liver of healthy animals that did not receive APAP (Fig. 3D, left photo). Seven days after intoxication, the necrotic areas were no longer present in the livers of the GStemHep-treated mice (Additional file 6: Fig. S4C). Therefore, frozen GStemHep rescue mice from a massive and rapid death induced by APAP intoxication and massive hepatocyte death with a protective effect observed within 24 h and as soon as 3 h post-cell transplantation.

### GStemHep tracking in APAP-induced acute liver failure

**Fig. 4:**
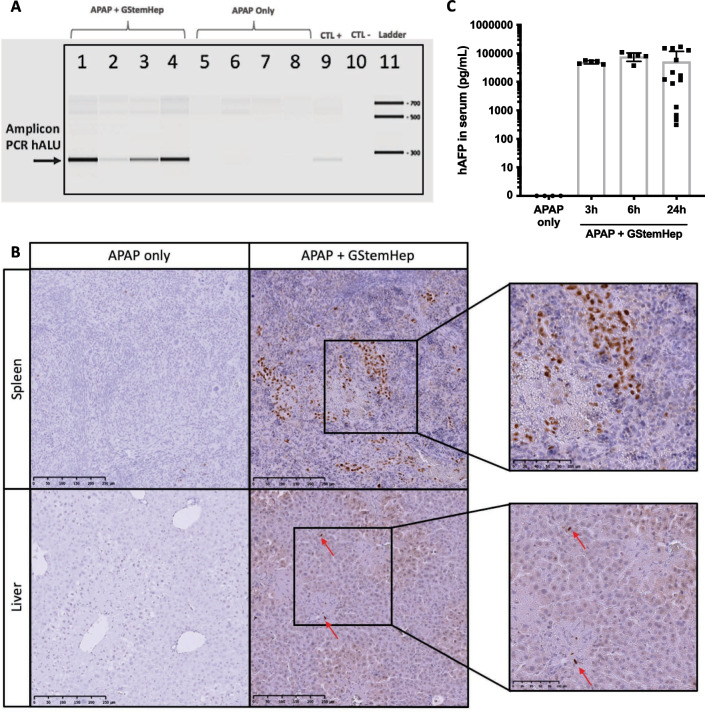
GStemHep tracking in the APAP-ALF model after transplantation. After APAP intoxication and cell transplantation (APAP + GStemHep), cell homing was analyzed in the liver and the spleen, and human AFP were quantified in the serum of mice at different times post-treatment (3 h, 6 h and 24 h). Untreated APAP-ALF mice served as controls (APAP only). **A** Detection of human ALU DNA sequences (human specific/290 bp) in mouse liver by PCR at 24 h after transplantation; each number represents a different mouse. **B** Immuno-histochemical staining for specific human Ku80 in mouse liver and spleen at 24 h after transplantation (Magnification 10 × on the left and 20 × on the right), data representative of 10 analyzed mice. (C) Quantification of human AFP in mouse serum by ELISA 3 h, 6 h and 24 h after transplantation (n = 4 in APAP only, n = 5 in APAP + GStemHep at 6 h and 9 h, n = 14 in APAP + GStemHep at 24 h post-transplantation), each point represents a different mouse

To demonstrate the presence of GStemHep in the liver, we performed a targeted amplification of human DNA in animal samples. Targeted PCR amplification of the human ALU sequence demonstrated the specific presence of cells in the liver of GStemHep-treated mice 24 h after transplantation (Fig. 4A). We confirmed that GStemHep homed to the livers using human Ku80 immunohistochemical staining of the liver of GStemHep-treated mice at 24 h post-transplantation (Fig. 4B). GStemHep cells were also detected in the spleen, as expected due to intrasplenic injection. Most of the cells are detected in the spleen at this time. Human *ALU*-PCR analyses in liver, spleen and lungs were negative at 7 days post-transplantation (Additional file 7: Fig. S5A).

Human AFP was detected in mouse serum at 3 h, 6 h and 24 h after cell transplantation, confirming that the thawed GStemHep cells were rapidly functional in vivo upon thawing and administration (Fig. 4C). The mean level of human AFP in mice serum was not significantly different between the 3 time points and ranged from 49.1 ng/mL ± 2.9 at 3 h post-cell transplantation to 78.3 ng/mL ± 11.6 at 6 h post-transplantation. The quantity of human AFP at 24 h post-cell transplantation was more heterogeneous between GStemHep-treated mice (52.1 ± 17.6 ng/mL).

### GStemHep lead to decreased inflammation and the restoration of liver functions.

**Fig. 5:**
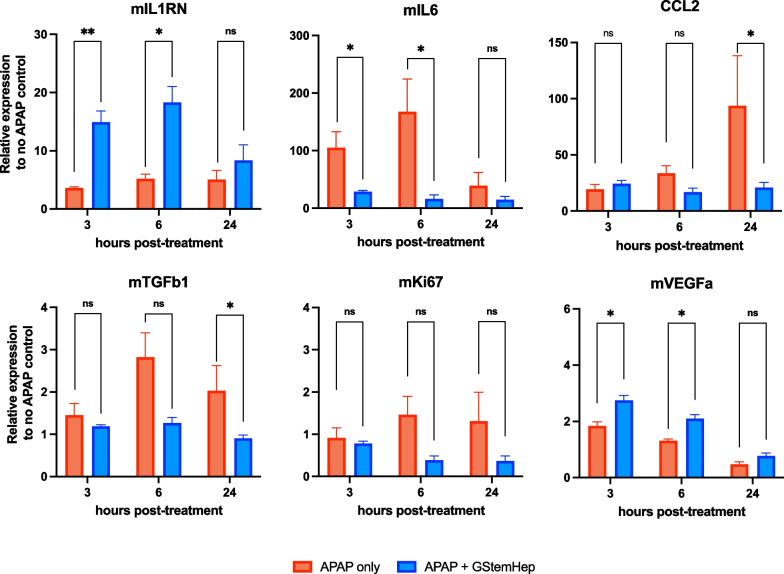
Mechanistic effects of GStemHep in APAP-ALF NOD/SCID mice. After APAP intoxication, mice were treated (APAP + GStemHep) or not (APAP only) with 1 × 10^6^ thawed GStemHep. Mouse livers were collected at 3 h, 6 h and 24 h post cell transplantation to analyze variations in gene expression. RT‒qPCR analysis of the expression of inflammation or regeneration markers (mIL1RN, mIL6, mCCL2, mTGFβ, mKi67 and mVEGFa) in the APAP only and APAP + GStemHep groups (n = 5 per group). The results were normalized to the mGAPDH housekeeping gene and expressed as the fold change relative to healthy control mice (*p < 0.05; **p < 0.01; ns: not significant, Mann‒Whitney test)

To better understand the therapeutic mechanisms of GStemHep in the APAP-induced ALF model, the livers of APAP mice with or without treatment were collected at 3 h, 6 h and 24 h post cell transplantation, analyzed and compared to healthy mouse livers. The inflammation and proliferation liver profiles were first analyzed by RT‒qPCR (Fig. 5). The mouse interleukin-1 receptor antagonist (IL1RN gene), a potent anti-inflammatory antagonist of IL-1 family cytokines, which are key inflammatory cytokines in ALF development [34], was statistically increased in the GStemHep-treated group in the short term (3 h and 6 h) compared to untreated ALF mice. The overexpression of the proinflammatory cytokine IL6, another hallmark of ALF, was reduced at 3 h and 6 h in GStemHep-treated ALF mice compared to untreated ALF mice. Of note, the mouse IL-6 level was still significantly detected in the treated ALF mice above the basal level of control mice with no APAP. The expression of chemokine ligand 2 (CCL2), involved in inflammation during ALF [35], was significantly reduced 24 h after transplantation. Other inflammatory molecules (TNFα, IL1β and ICAM1) were studied but showed no significant difference between GStemHep-treated ALF mice and untreated ALF mice (Additional file 8: Fig. S6A). Twenty-four hours after transplantation, TGFβ1 was significantly decreased in animals treated with GStemHep. The expression of the mouse Ki67 proliferation marker did not differ between the GStemHep-treated and untreated groups. This observation was also confirmed by immunohistochemical labeling of Ki67 in liver tissues (Additional file 9: Fig. S7). Interestingly, the gene expression of mouse vascular endothelial growth factor A (VEGFa) was significantly increased in the GStemHep-treated groups at 3 h and 6 h post-cell transplantation.

**Fig. 6:**
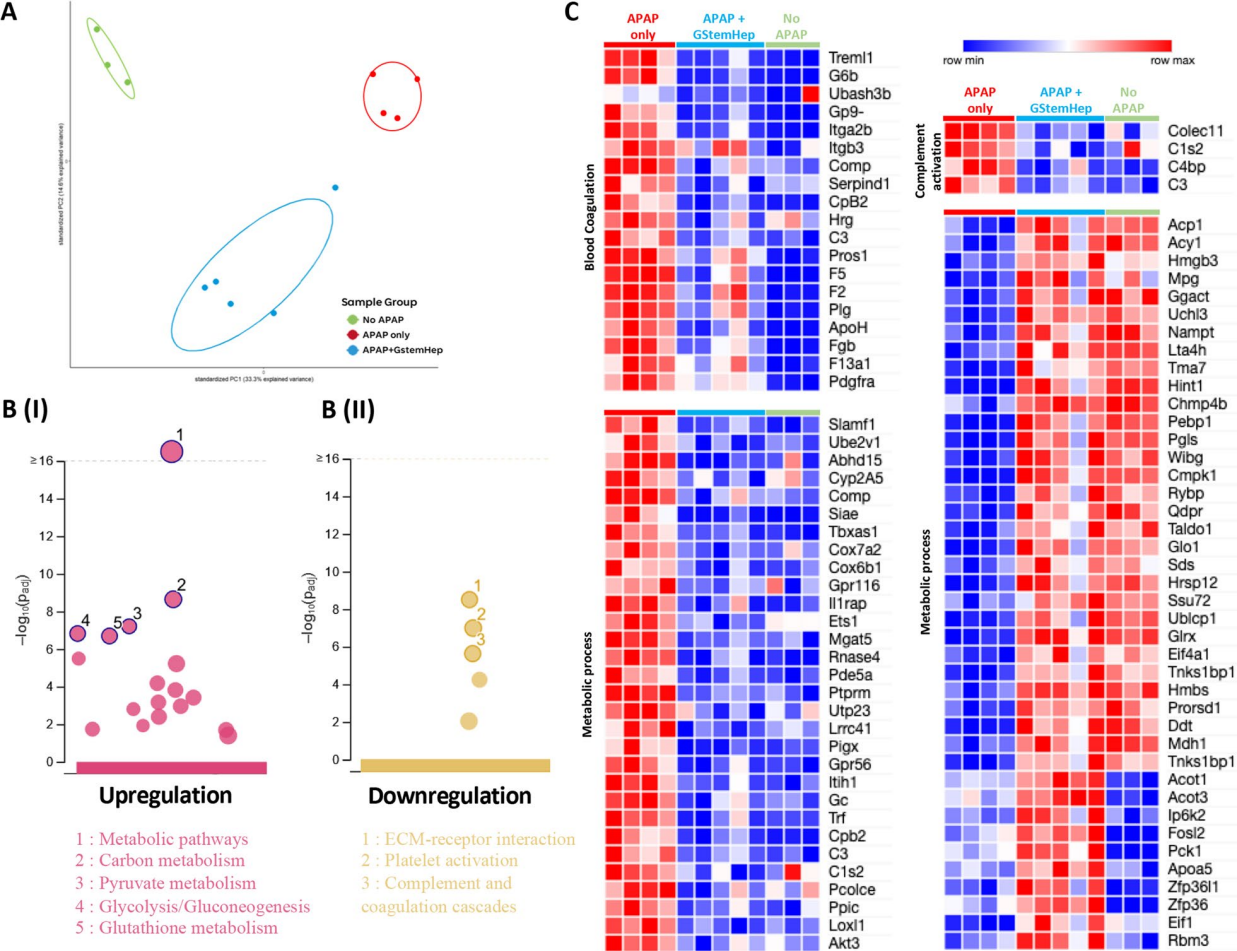
Mechanistic effects of GStemHep in APAP-ALF NOD/SCID mice. After APAP intoxication, mice were treated (APAP + GStemHep) or not (APAP only) with 1 × 10^6^ thawed GStemHep. Mouse livers were collected at 6 h post cell transplantation to analyze variations in protein expression. **A** Proteome-wide data set variance described by Principal Component Analysis (PCA) on healthy (no APAP, n = 3), APAP only (n = 4) and APAP + GStemHep groups (n = 5), 6 h after cell transplantation (i.e., 9 h post-APAP intoxication). **B** KEGG pathway analysis of differentially regulated (up- and downregulated) proteins in the APAP + GStemHep group compared to the APAP group. (I) Upregulation pathways in pink. (II) Downregulation pathways in yellow. **C** Heatmap visualizing the intensities of differentially regulated proteins in three GO annotations (blood coagulation, complement activation and metabolic process) between all groups; each line represents a protein

The therapeutic effect of GStemHep was then evaluated by proteomic analysis using HRM™ mass spectrometry of liver samples from GStemHep-treated ALF mice at 6 h post cell transplantation (APAP + GStemHep), untreated control ALF mice at time corresponding to 6 h post-GStemHep cell transplantation (APAP only) and healthy control mice (no APAP). Principal component analysis (PCA) was performed to visualize data set variance in dependence on sample groups (Fig. 6A). PCA showed clear separation between the three mouse groups, with the main variance PC1 and the secondary variance PC2, explained by differences between the sample groups and by the effect of treatment, respectively. The number of differentially regulated proteins between the healthy mouse group and the untreated ALF mouse group was 861. In contrast, this number was lower between the healthy mouse group (WT group) and the GStemHep-treated ALF mouse group with 275 differentially regulated proteins, showing that treated ALF mice were closer to healthy mice than untreated ALF mice. There were 320 differentially regulated proteins between the GStemHep-treated and untreated ALF mouse groups. If we focus on the proteins that are specifically differentially expressed among the three groups in the Venn diagram, we again find a large number of proteins (524) in the APAP only / WT comparison versus 121 and 109 between the APAP + GStemHep / APAP only and APAP + GStemHep / WT groups, respectively (Additional file 8: Fig. S6B). KEGG pathway analysis of these differentially regulated proteins showed 23 KEGG pathways enriched in the GStemHep-treated group (Fig. 6B). Metabolic pathways were the most representative in upregulation, including carbon metabolism, glycolysis/gluconeogenesis, pyruvate and glutathione metabolism (Fig. 6BI). At variance, 5 pathways were downregulated, including ECM-receptor interaction, platelet activation and complement and coagulation cascades (Fig. 6BII). Finally, the gene ontology (GO) function annotation of differential proteins showed three GO annotations of differentially regulated proteins represented in heatmaps (Fig. 6C). The proteins involved in coagulation and complement activation were upregulated in the APAP-only group. After treatment with GStemHep cells, these proteins returned to healthy values. Concerning metabolic processes, all proteins that were upregulated after intoxication returned to the basal level of the healthy group after treatment. Of the downregulated metabolic process proteins after intoxication, 73% returned to the basal expression of the healthy group after treatment.

Thus, after GStemHep treatment, the function of host hepatocytes appears to recover, and APAP-induced inflammation declines.

### Frozen GStemHep rescue TAA-induced acute liver failure in immunocompetent mice

**Fig. 7:**
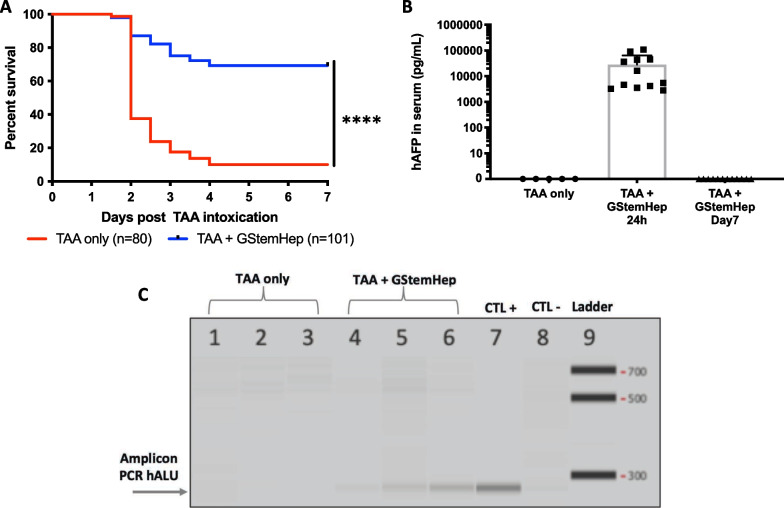
Therapeutic effect of GStemHep and cell tracking in C57Bl/6 mice with TAA-induced ALF. After TAA intoxication, mice were treated (TAA + GStemHep) or not (TAA only) with intrasplenic injection of 1 × 10^6^ GStemHep. Mouse survival was monitored for 7 days, or mice were euthanized at 24 h post treatment for PCR analysis of the liver. Sera were collected at different times. **A** Survival curve of TAA-induced ALF mice treated with GStemHep (n = 101) compared with non-treated group (n = 80) (****p < 0.0001 log-rank (Mantel‒Cox) test). **B** Quantification of human AFP in the serum of the TAA-only group (n = 5) and the TAA + GStemHep group at 24 h (n = 13) and 7 days (n = 5) after GStemHep transplantation by ELISA. **C** Detection of human ALU DNA sequences in mouse livers by PCR at 24 h after transplantation; each number represents a different mouse

To confirm and further highlight the therapeutic potential of GStemHep for ALF, we used a second well-described ALF mouse model in immunocompetent C57Bl/6 animals lethally intoxicated with TAA hepatotoxin. Twenty-four hours after TAA intoxication, animals received an intrasplenic injection of thawed GStemHep in the absence of an immunosuppression drug. Immediately after cell thawing and without any cell wash step, GStemHep cells were injected into the portal vein via the intrasplenic administration route. We observed, as for the APAP model, a significantly larger survival of transplanted mice (69%) compared to untreated mice (10%) (*p* < 0.0001) (Fig. 7A). This was demonstrated using 6 different cell production batches (cryopreserved between 2 and 9 months) in 20 different animal studies, with n = 181 animals showing both the robustness of the cell production process and the therapeutic efficacy of GStemHep. Interestingly, 83% of untreated ALF control mice died within 48 h after transplantation, showing that cell therapy needed to be efficient within the short term to rescue mice from ALF-induced death (Fig. 7A).

Human AFP could be detected in mice serum for 24 h (28 ± 10 ng/mL on average). Notably, human AFP in mice serum was not detected after 7 days (Fig. 7B). Human *ALU* PCR analyses showed the presence of human DNA in the mouse livers 24 h after cell transplantation (Fig. 7C) but not in the liver, spleen or lungs at 9 days (Additional file 7: Fig. S5B).

## Discussion

The encouraging results of hepatocyte transplantation and the emergence of PSC as an unlimited cell source have paved the way for a new cell therapy for ALF. The challenges of this therapy are the production of hepatic cells cultured and differentiated in a cGMP-compliant manner and on a large scale while maintaining therapeutic potency. In view of the emergency, it is also essential to guarantee the availability of the treatment at any time in the clinical context.

In this study, we investigated the potential therapeutic effect of frozen human PSC-derived hepatic progenitors called GStemHep, produced in cGMP-compliant feeder-free and serum-free conditions. Indeed, at present, very few proposed protocols are fully cGMP, i.e., for both PSC amplification and hepatocyte differentiation steps [13]. Several cGMP-compliant hepatic differentiation protocols have been described, but PSCs were generally amplified using non-cGMP matrices for PSC culture, such as Matrigel [19, 20, 25]. Here, all raw materials, media, cytokines and matrix used for PSC amplification and differentiation are compatible with cGMP standards.

In addition, all previous studies were focused on the therapeutic potential of PSC-derived HLCs, i.e., similar to fetal and not fully differentiated hepatocytes [13–17, 20]. The HLCs were characterized mainly by the expression of the fetal marker AFP, the mature hepatic marker ALB and significant detoxification activities (CYP3A4, ammonia detoxification, etc.). They showed therapeutic potential when transplanted in animal models with various liver failures, including ALF [14, 20–22, 36, 37]. In our study, we demonstrate that immature hepatic progenitors (AFP + /ALB−) with no detoxification activities at the time of transplantation show therapeutic effects for treating ALF. Noteworthy, the duration of the differentiation protocol of GStemHep is significantly shorter (10 days) than that of HLC (> 20 days), resulting in cost and time savings. Interestingly, GStemHep became less immunogenic after hepatic differentiation (loss of expression of MHC class I and no expression of MHC class II).

The other two major limitations of PSC-based therapeutic treatment include the need for sufficient quantities of cells to be available at all times. Based on the number of cells used in successful clinical trials involving primary hepatocytes in ALF [5] and the extrapolation of our preclinical data, the expected clinical dose of GStemHep is 1 to 2 billion per patient. This cell quantity requires large-scale production of PSC-derived cells. The feasibility of large-scale production has already been demonstrated previously [19, 38–40]. In addition, Goliver Therapeutics has demonstrated proof-of-concept results for PSC amplification and two-dimensional production of GStemHep under GMP conditions in quantities sufficient for a phase I/IIa clinical trial (patient doses of 1 or 2 billion cells). Cell cryopreservation allows immediate disposal at any time, particularly for emergency use, thus addressing the second issue. However, for now, all the various studies showing a therapeutic effect of HLC in ALF animal models used freshly prepared cells [19, 20, 41–43]. Indeed, cryopreservation and thawing procedures have been reported to have detrimental effects on the viability and function of human hepatocytes when compared to freshly isolated cells [23, 24]. Improved hepatocyte cryopreservation protocols have been described, but therapeutic efficacy has not yet been reported in ALF [43, 44]. We previously reported that frozen PSC-derived hepatic stem cells were able to rescue Crigler-Najjar inherited disease mice, i.e., in the context of non-fulminant liver diseases where transplanted cells have time to differentiate into fully mature hepatocytes [25]. The question that remains is whether frozen cells can save liver failure outside of inherited metabolic diseases in an acute and inflammatory context. Here, the overall freezing conditions of GStemHep were optimized for high viability after thawing with maintenance of the phenotype and the adhesion capacities of freshly produced cells. Furthermore, frozen GStemHep displayed significant therapeutic potential after thawing and immediate transplantation without prior cell washing steps in two ALF mouse models. Their resistance to cryopreservation-related damage may be associated with the immature hepatic phenotype of the cells (AFP + /ALB−).

The therapeutic effect resulted in a significant increase in the survival of ALF mice and a decrease in hepatic injury markers (transaminase, liver necrosis), which was observed as soon as 3 h post-cell transplantation in the APAP-induced ALF mouse model. This showed that the action of GStemHep is very rapid. Moreover, the therapeutic effect was observed in both partially immunodeficient and immunocompetent models in the absence of immunosuppression, where xenogeneic immune rejection is stronger than in allogeneic human clinical settings. The in vivo therapeutic potential was demonstrated in several independent animal experiments (8 for APAP-ALF and 20 for TAA-ALF mice) and using different cell batches (up to 6), showing both the robustness of the therapeutic efficacy of GStemHep and the cell production process. Interestingly, the therapeutic efficacy of GStemHep seems to be greater than or at least equivalent to that of primary human hepatocytes (100 vs. 60% survival, but not significantly different), which confirms that immature hepatic progenitors (AFP + ALB−) can be therapeutic for acute liver failure.

We showed that GStemHep became rapidly functional upon thawing as soon as 3 h post-transplantation (presence of human AFP in mouse serum) and homed to the liver within 24 h (hAlu-PCR, anti-hKu80 immunostaining). GStemHep can be detected as soon as 3 h post-transplantation in the liver after the splenic administration route and disappears within 7 days. A more precise exploration of biodistribution throughout the organism (by bioluminescent labeling of GStemHep, for example) should be carried out before clinical studies. However, the absence of long-term cell engraftment in the liver, the very rapid death of mice and the rapidity of the therapeutic effect suggest a paracrine mechanism of action of GStemHep.

We demonstrated that GStemHep produced and secreted a significant level of HGF, which is the most potent growth factor for hepatocytes [22] and has anti-inflammatory activity [45, 46]. This secreted HGF probably plays a major role in the therapeutic effects of GStemHep. Indeed, several studies have shown that the production of HGF by HLC or mesenchymal stem cells (MSC) is essential for rescuing ALF mice [22, 43, 47]. However, Wang et al. also demonstrated that, despite its role in the therapeutic effect of MSC, the injection of HGF alone was not sufficient to significantly increase the survival of ALF-APAP mice, in contrast to our results with GStemHep [47]. This finding suggested that there are likely additional therapeutic molecules secreted by GStemHep. A more exhaustive study using proteomics and cytokine profiling, including exosome studies coupled with knockdown studies using multiple siRNAs, should enable us to more precisely describe the complete modes of action of GStemHep.

To better understand the mode of action of GStemHep, unbiased proteomic and gene expression analyses were carried out on animal liver. Due to the rapid observed action of GStemHep, the expression of hepatic inflammation and regeneration genes was analyzed 3 h, 6 h and 24 h after treatment. Inflammation is reported to play a key role in liver failure and regeneration, but this role must be finely regulated [48]. Tuning this response could be as important as supporting impaired organ functions. Despite its role in liver regeneration, excess inflammation in ALF leads to more serious damage [49]. Interestingly, we observed an increase in IL1RN and a decrease in IL6 gene expression as soon as 3 h post-cell transplantation and thereafter suggested that GStemHep may prevent an early excess of inflammation, a hallmark in ALF. For instance, decreasing IL6-related inflammation has been shown to restore cerebral blood flow and reduce features of hepatic encephalopathy in mice with APAP-induced ALF [50] and decrease liver damage [51, 52]. Notably, IL6 gene expression was still detected in GStemHep-treated mice over the basal level of healthy mice, which may be crucial for promoting liver regeneration of the injured liver. Concomitantly, we observed an increase in VEGFa gene expression at 3 and 6 h after treatment. As several reports have demonstrated an important role of VEGFa in liver regeneration and hepatocyte proliferation after APAP-induced hepatotoxicity [53–55], enhanced expression of VEGFa could contribute to the therapeutic effect of GStemHep. No effect on hepatocyte proliferation was observed within 24 h post-cell transplantation. With peak hepatocyte proliferation after major partial hepatectomy occurring at 48 h [56] and 72 h after high-dose APAP [57], the analysis of proliferation markers (Ki67) in this study was therefore too early to show a significant increase in liver regeneration after GStemHep treatment.

Unbiased proteomic analyses of liver samples confirmed the different expression profiles between treated and untreated APAP-induced ALF mice. The primary analysis of the liver proteome at 6 h after cell transplantation also suggests a return toward the basal state of the healthy liver. Indeed, there is a greater expression of the proteins involved in the metabolic pathways of the liver in the treated mice, suggesting a more functional liver compared to untreated ALF mice. At the same time, proteins involved in the complement and coagulation activation cascades were significantly reduced with treatment. Activation of coagulation and platelet accumulation contribute to liver cell damage during APAP intoxication [58, 59]. These results therefore suggest a cytoprotective effect of the cells.

Noteworthy, proteomic results at 6 h post-GStemHep treatment revealed that TGFβ1, which is known to inhibit hepatocyte proliferation after partial hepatectomy and to inhibit liver regeneration after APAP toxicity, is increased in untreated APAP-ALF mice but significantly decreased in GStemHep-treated APAP-ALF mice to a similar level to that of healthy mice [60]. These observations were confirmed by qPCR analysis of TGFβ1 gene expression. Concomitantly, we observed a significant increase in the expression of WNT/βcatenin in GStemHep-treated mice compared to untreated APAP-ALF mice. It has been shown that increased expression of the WNT/βcatenin signaling pathway improves liver regeneration after APAP overdose [57]. Deeper data analyses are underway to more precisely define all the molecules and biological pathways that ultimately mediate liver protection from ALF by GStemHep.

The final aspect we need to explore in greater depth is biosafety. Indeed, certain properties of cell therapies, such as differentiation and proliferation potential, raise safety issues. These cellular products carry risks associated with localized host tissue reactions, differentiation into undesirable cell and tissue types, uncontrollable biodistribution, tumorigenicity and immunogenicity [61]. Tumorigenicity studies in an in vivo model in NODSCID mice over 3 months and an in vitro soft agar model [62] were carried out (not shown). GStemHep do not present any risk of tumorigenicity in these tests, but biosafety studies are essential and remain in progress.

## Conclusion

In conclusion, we describe the production of GStemHep, which are immature hepatic cells (AFP + /ALB−) secreting HGF and derived from human pluripotent stem cells, using fully cGMP-compliant protocols. We show a clear and rapid therapeutic effect in different models of ALF after injection of a single dose of GStemHep, demonstrating that therapeutic potential is not bound to a mature hepatic phenotype. These GStemHep cells can be cryopreserved and used after thawing without impacting their therapeutic potential. They have a rapid therapeutic effect, as soon as 3 h post-cell transplantation, with a decrease in liver injuries and an increase in metabolic functions. Their mode of action relies on alleviation of inhibition factors of liver regeneration, increase in proliferation-promoting factors and decrease in liver inflammation. Finally, their therapeutic effects can be demonstrated in immunocompetent animals in the absence of immunosuppression. Overall, these results pave the way to conduct a phase I/II clinical trial for PSC-based regenerative medicine for acute liver failure with a single dose of frozen GStemHep in the absence of immunosuppression to repair the liver without grafts.

### Supplementary Information

**Additional file 1**. **Table S1**: Primers for Taqman assays

**Additional file 2**. **Table S2**: Antibodies for Immunofluorescence Assay

**Additional file 3**. **Figure S1**. Immune profile of GStemHep. Quantification of MHC class I (HLA-ABC) and class II (HLA-DR) molecules in the PSC cell line and GStemHep production batches (n=8, each dot represents a cell batch) by flow cytometry

**Additional file 4**. **Figure S2**. Cryopreservation of GStemHep. After 10 days of differentiation, GStemHep cells were harvested and frozen in a cryopreservation solution. The hepatic phenotype was analyzed after thawing on one production. (A) RT‒qPCR and (B) FACS analysis of key marker expression in a cell production batch before and after freezing

**Additional file 5**. **Figure S3**. Therapeutic effect of GStemHep compared to primary that of human hepatocytes in NOD/SCID mice with APAP-induced acute liver failure. After APAP intoxication, the mice were treated with 1x10^6^ thawed GStemHep or primary hepatocytes, or not (APAP only). Survival curve of mice followed for more than 10 days (ns: not significant; **p<0.005 log-rank (Mantel‒Cox) test)

**Additional file 6**. **Figure S4**. Therapeutic effects of GStemHep in NOD/SCID mice with APAP-induced ALF. (A) Biochemical analysis of liver damage markers: ASAT and ALAT in blood serum of each group at 3 h and 6 h after cell transplantation (* p<0.05; ** p<0.005; ***p<0.0005, one-way ANOVA test) (n=5 in each group). (B) Representative HES-stained sections of liver at 3 h after APAP injection, i.e., at the time and before GStemHep treatment and (C) 7 days after cell transplantation (Magnification x1 on the left and x5 on the right), data representative of 5 analyzed mice at each time point.

**Additional file 7**. **Figure S5**. GStemHep tracking in the APAP- and TAA-induced ALF models long-term post-transplantation. (A) Detection of ALU DNA sequences (human specific/290 bp) in mouse liver, spleen and lungs by PCR at 7 days after GStemHep transplantation in APAP-ALF mice. (B) Detection of ALU DNA sequences (human specific/290 bp) in mouse liver, spleen and lungs by PCR at 9 days after transplantation in TAA-ALF mice. Each number represents a different mouse (1-4: APAP + GStemHep; 5-7: TAA + GStemHep; C+: positive control; C-: negative control).

**Additional file 8**. **Figure S6**. Mechanistic effects of GStemHep in APAP-ALF NOD/SCID mice. After APAP intoxication, the mice were treated (APAP+GStemHep) or not (APAP only) with 1x10^6^ thawed GStemHep. Mouse livers were collected at 6 h post cell transplantation to analyze variations in gene and protein expression. (A) RT‒qPCR analysis of the expression of inflammatory markers (mTNFα, mIL1β, and mICAM1) in the APAP only and APAP+GStemHep groups (n=5 per group). The results were normalized to the mGAPDH housekeeping gene and expressed as the fold change relative to healthy control mice (ns: not significant, Mann‒Whitney test). (B) Venn diagram illustrating the overlap of differentially regulated proteins between the three groups comparisons analyzed by mass spectrometry.

**Additional file 9**. **Figure S7**. Evaluation of cell proliferation in the liver of APAP-ALF NOD/SCID mice after GStemHep treatment. (A) Immunohistochemical staining images for the proliferation marker Ki67 in the livers of healthy (no APAP), untreated APAP-ALF (APAP only) and GStemHep-treated (APAP + GStemHep) mice at 24 h after cell therapy (magnification 10x); data are representative of 5 analyzed mice per group. (B) Quantification of the Ki67+ area in the livers of untreated APAP-ALF (APAP only) and GStemHep-treated (APAP + GStemHep) mice (n=5; ns: not significant, Mann‒Whitney test).

## Data Availability

The mass spectrometry proteomics data have been deposited to the ProteomeXchange Consortium via the PRIDE [63] partner repository with the dataset identifier PXD049134.

## Abbreviations

AAT: Alpha-1 antitrypsin
AFP: Alpha-fetoprotein
ALAT: Alanine amino-transferase
ALB: Albumin
ALF: Acute liver failure
ANOVA: Analysis of variance
APAP: Acetaminophen
ASAT: Aspartate amino-transferase
BSA: Bovine serum albumin
CCL2: Chemokine ligand 2
cGMP: Current good manufacturing practice
CK19: Cytokeratin 19
CYP: Cytochrome
DAB: 3,3' Diaminobenzidine
DNA: Deoxyribonucleic acid
EDTA: Ethylenediaminetetraacetic acid
FACS: Fluorescence-activated cell sorting
FOXA2: Forkhead box A2
GAPDH: Glyceraldehyde 3-phosphate dehydrogenase
GO: Gene ontology
HES: Hematoxylin-eosin-saffron
HGF: Hepatocyte growth factor
HLA: Human leukocyte antigen
HLC: Hepatocyte-like cells
HNF1β: Hepatocyte nuclear factor 1 beta
HNF4: Hepatocyte nuclear factor 4
HRM: Hyperreaction monitoring
HRP: Horseradish peroxidase
HT: Hepatocyte transplantation
ICAM1: Intercellular adhesion molecule 1
IHC: Immunohistochemistry
IL: Interleukin
IP: Intraperitoneal
KEGG: Kyoto Encyclopedia of Genes and Genomes
MHC: Major histocompatibility complex
MSC: Mesenchymal stem cells
NOD/SCID: Non obese diabetic/severe combined immunodeficiency
OCT4: Octamer-binding transcription factor 4
OLT: Orthotopic liver transplantation
PCA: Principal component analysis
PSC: Pluripotent stem cells
RNA: Ribonucleic acid
RT‒qPCR: Real-time quantitative polymerase chain reaction
SOX17: SRY-box transcription factor 17
TAA: Thioacetamide
TBX3: T-box transcription factor 3
TGFβ1: Transforming growth factor beta 1
TNFα: Tumor necrosis factor alpha
VEGFa: Vascular endothelial growth factor A
WT: Wild type
