# Supplementary figures and images for: Cryopreserved cGMP-compliant human pluripotent stem cell-derived hepatic progenitors rescue mice from acute liver failure through rapid paracrine effects on liver cells
Source: Stem Cell Res Ther. 2024 Mar 12;15:71. doi: 10.1186/s13287-024-03673-9 (PMC10935817; doi:10.1186/s13287-024-03673-9)

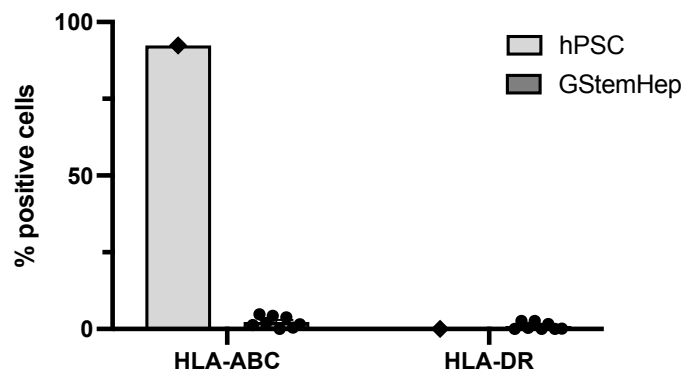

Supplement: Supplementary file 3 — Additional file 3. Figure S1. Immune profile of GStemHep. Quantification of MHC class I (HLA-ABC) and class II (HLA-DR) molecules in the PSC cell line and GStemHep production batches (n=8, each dot represents a cell batch) by flow cytometry [file 13287_2024_3673_MOESM3_ESM.pdf]

**A**

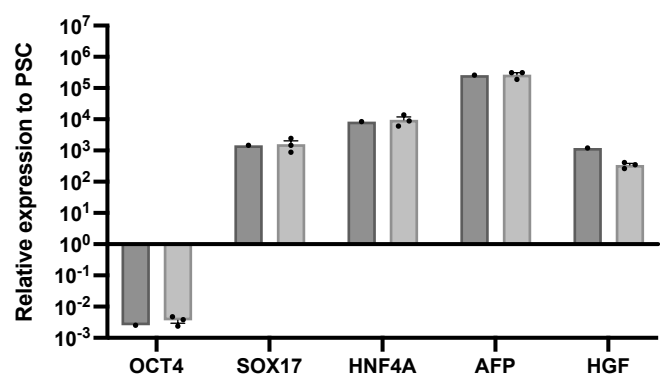

■ Fresh GStemHep    ■ Thawed GStemHep

**B**

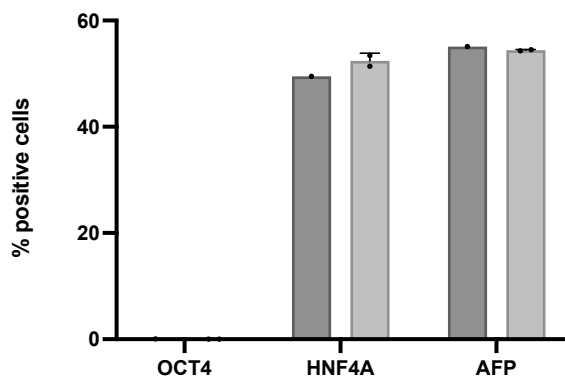

Supplement: Supplementary file 4 — Additional file 4. Figure S2. Cryopreservation of GStemHep. After 10 days of differentiation, GStemHep cells were harvested and frozen in a cryopreservation solution. The hepatic phenotype was analyzed after thawing on one production. (A) RT‒qPCR and (B) FACS analysis of key marker expression in a cell production batch before and after freezing [file 13287_2024_3673_MOESM4_ESM.pdf]

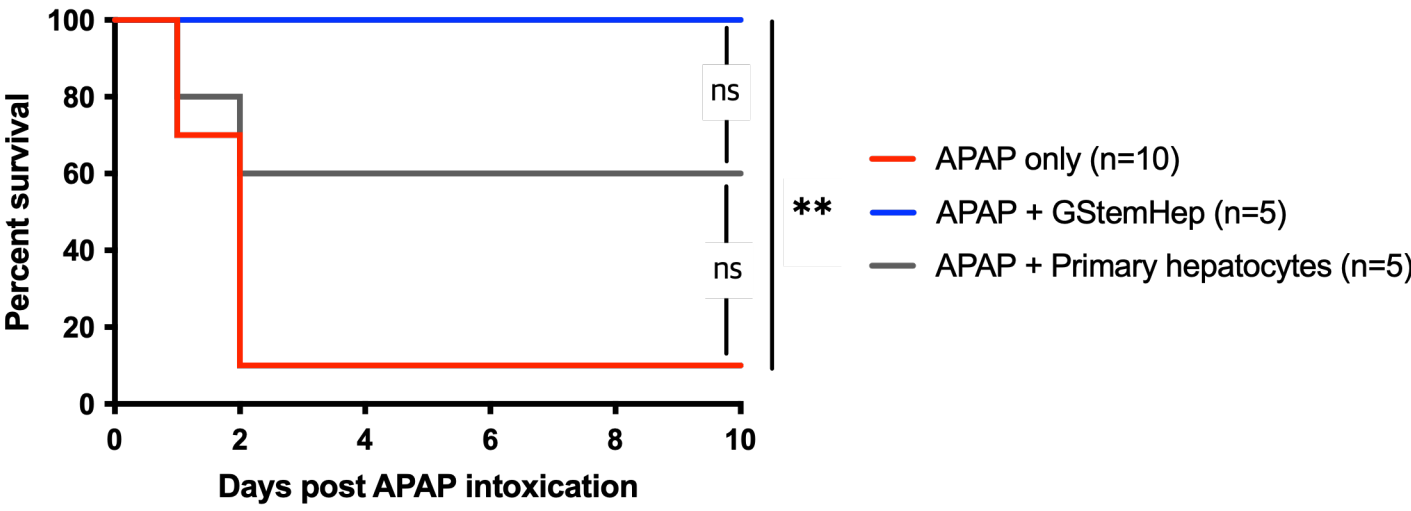

Supplement: Supplementary file 5 — Additional file 5. Figure S3. Therapeutic effect of GStemHep compared to primary that of human hepatocytes in NOD/SCID mice with APAP-induced acute liver failure. After APAP intoxication, the mice were treated with 1x10^6^ thawed GStemHep or primary hepatocytes, or not (APAP only). Survival curve of mice followed for more than 10 days (ns: not significant; **p<0.005 log-rank (Mantel‒Cox) test) [file 13287_2024_3673_MOESM5_ESM.pdf]

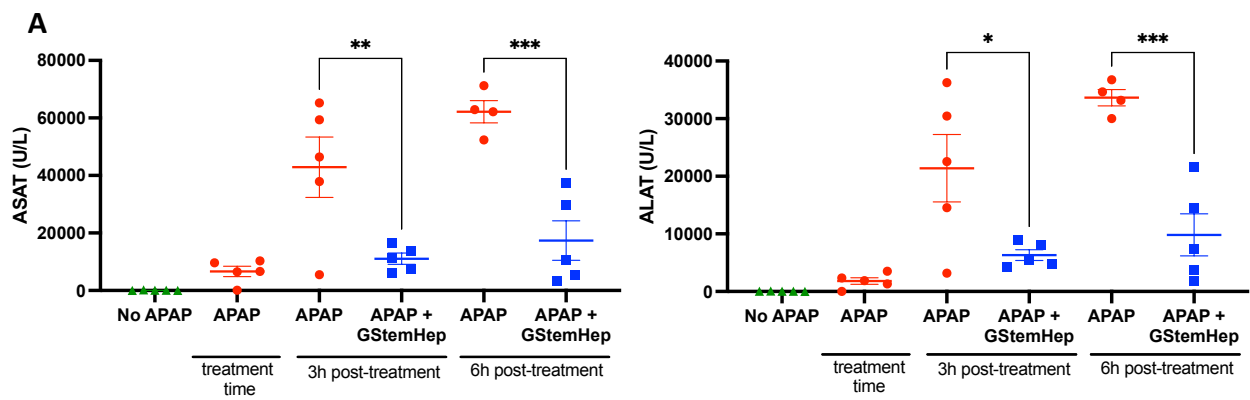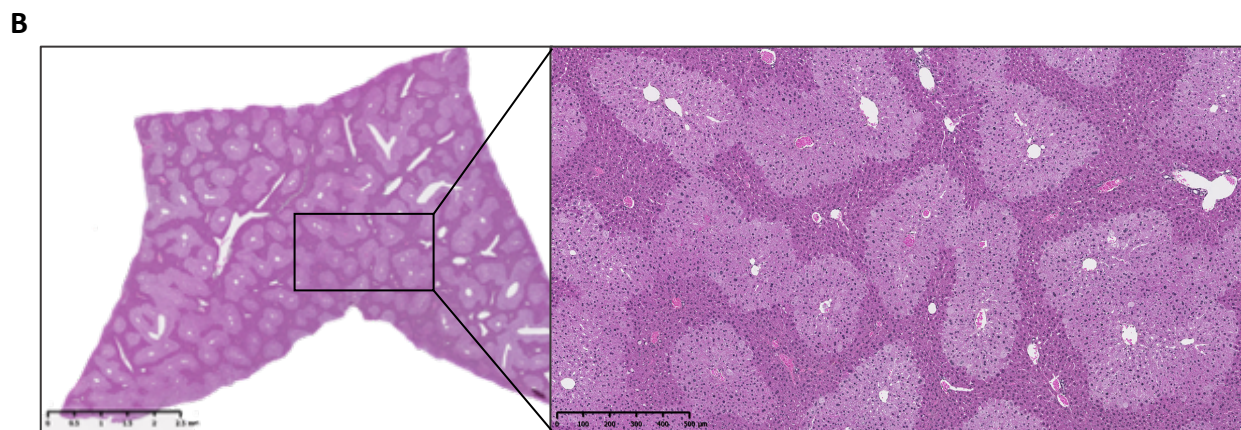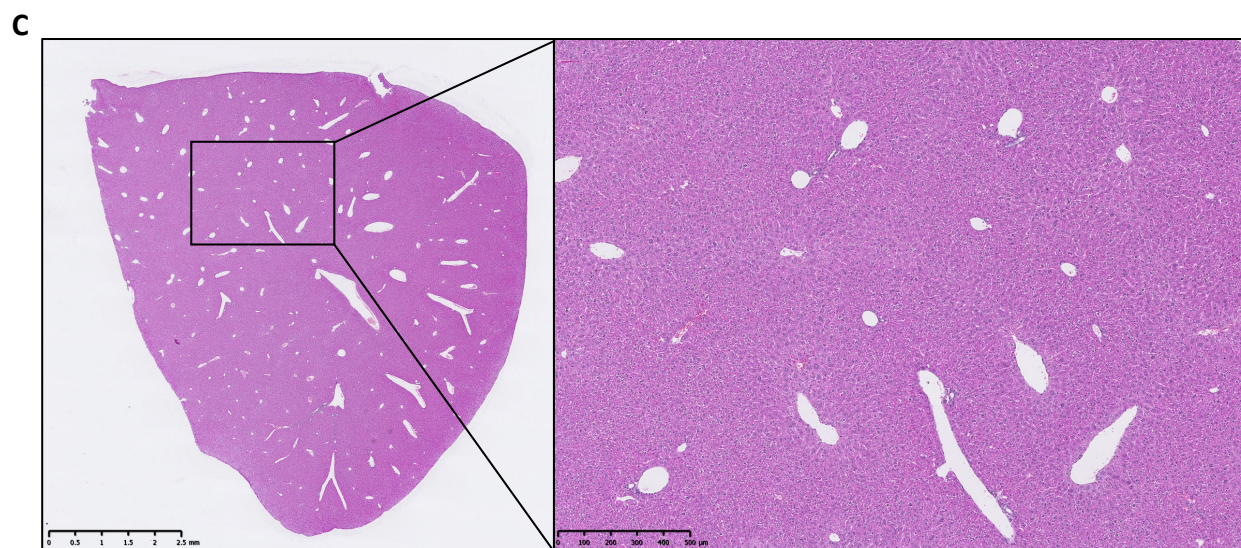

Supplement: Supplementary file 6 — Additional file 6. Figure S4. Therapeutic effects of GStemHep in NOD/SCID mice with APAP-induced ALF. (A) Biochemical analysis of liver damage markers: ASAT and ALAT in blood serum of each group at 3 h and 6 h after cell transplantation (* p<0.05; ** p<0.005; ***p<0.0005, one-way ANOVA test) (n=5 in each group). (B) Representative HES-stained sections of liver at 3 h after APAP injection, i.e., at the time and before GStemHep treatment and (C) 7 days after cell transplantation (Magnification x1 on the left and x5 on the right), data representative of 5 analyzed mice at each time point. [file 13287_2024_3673_MOESM6_ESM.pdf]

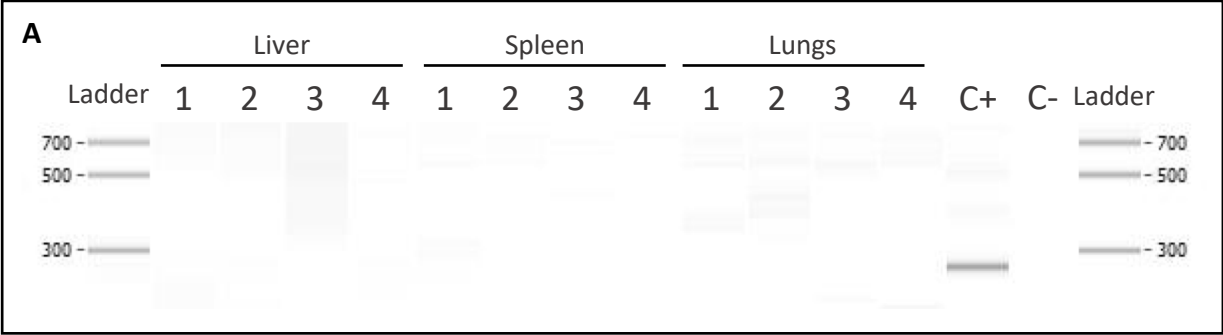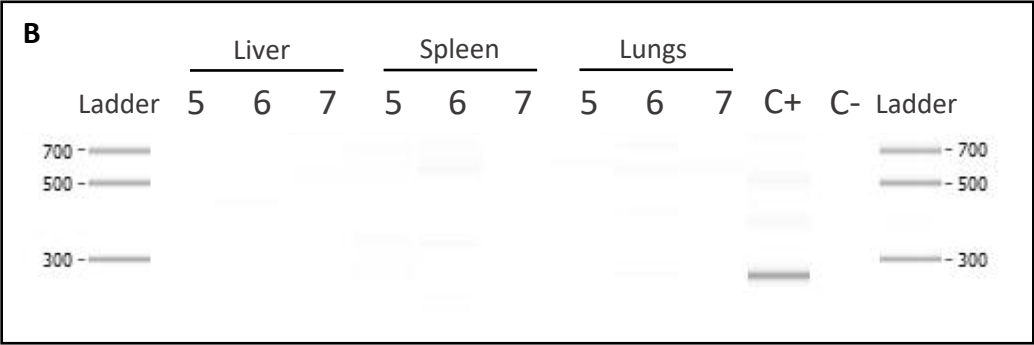

Supplement: Supplementary file 7 — Additional file 7. Figure S5. GStemHep tracking in the APAP- and TAA-induced ALF models long-term post-transplantation. (A) Detection of ALU DNA sequences (human specific/290 bp) in mouse liver, spleen and lungs by PCR at 7 days after GStemHep transplantation in APAP-ALF mice. (B) Detection of ALU DNA sequences (human specific/290 bp) in mouse liver, spleen and lungs by PCR at 9 days after transplantation in TAA-ALF mice. Each number represents a different mouse (1-4: APAP + GStemHep; 5-7: TAA + GStemHep; C+: positive control; C-: negative control). [file 13287_2024_3673_MOESM7_ESM.pdf]

A

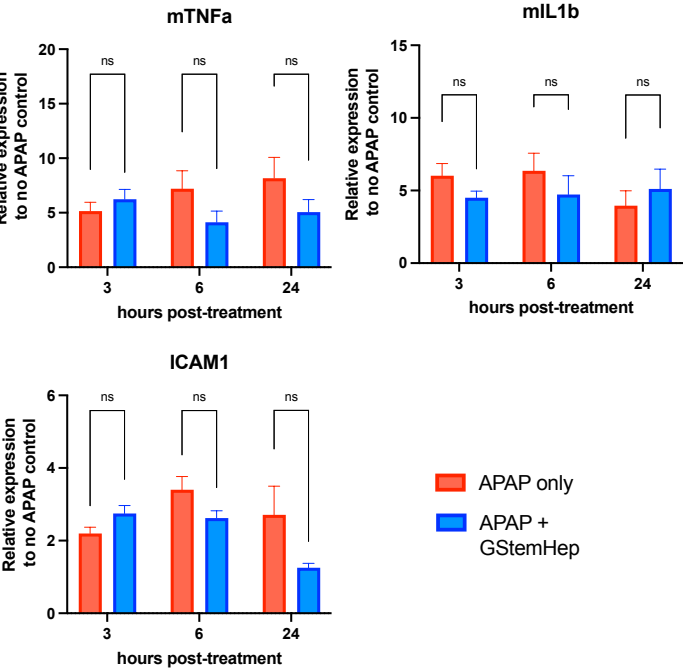

B

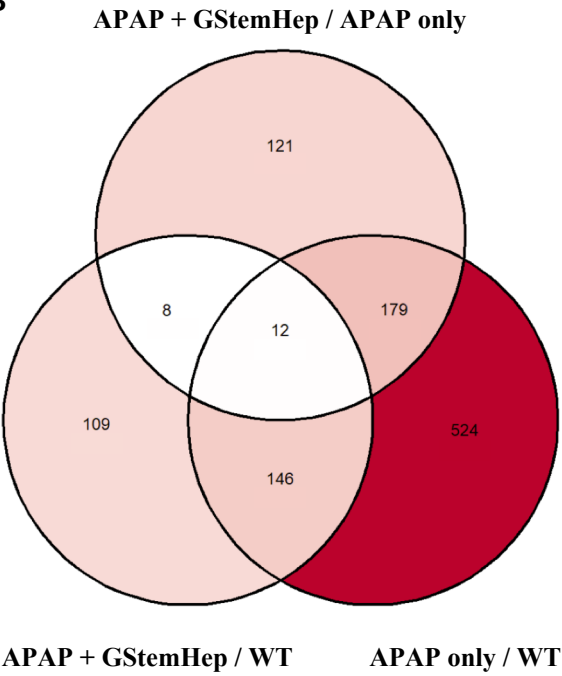

Supplement: Supplementary file 8 — Additional file 8. Figure S6. Mechanistic effects of GStemHep in APAP-ALF NOD/SCID mice. After APAP intoxication, the mice were treated (APAP+GStemHep) or not (APAP only) with 1x10^6^ thawed GStemHep. Mouse livers were collected at 6 h post cell transplantation to analyze variations in gene and protein expression. (A) RT‒qPCR analysis of the expression of inflammatory markers (mTNFα, mIL1β, and mICAM1) in the APAP only and APAP+GStemHep groups (n=5 per group). The results were normalized to the mGAPDH housekeeping gene and expressed as the fold change relative to healthy control mice (ns: not significant, Mann‒Whitney test). (B) Venn diagram illustrating the overlap of differentially regulated proteins between the three groups comparisons analyzed by mass spectrometry. [file 13287_2024_3673_MOESM8_ESM.pdf]

A

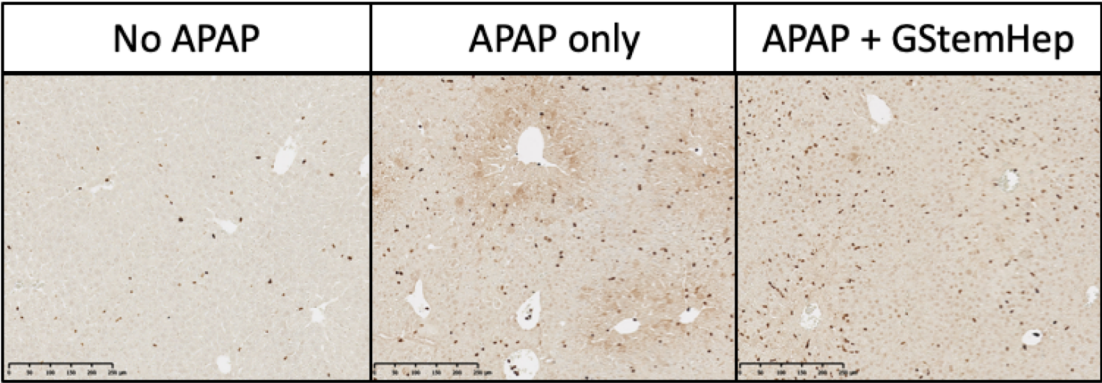

B

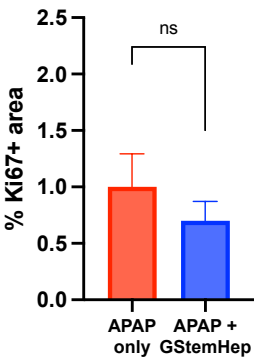

Supplement: Supplementary file 9 — Additional file 9. Figure S7. Evaluation of cell proliferation in the liver of APAP-ALF NOD/SCID mice after GStemHep treatment. (A) Immunohistochemical staining images for the proliferation marker Ki67 in the livers of healthy (no APAP), untreated APAP-ALF (APAP only) and GStemHep-treated (APAP + GStemHep) mice at 24 h after cell therapy (magnification 10x); data are representative of 5 analyzed mice per group. (B) Quantification of the Ki67+ area in the livers of untreated APAP-ALF (APAP only) and GStemHep-treated (APAP + GStemHep) mice (n=5; ns: not significant, Mann‒Whitney test). [file 13287_2024_3673_MOESM9_ESM.pdf]
